# ETMR: a tumor entity in its infancy

**DOI:** 10.1007/s00401-020-02182-2

**Published:** 2020-06-29

**Authors:** Sander Lambo, Katja von Hoff, Andrey Korshunov, Stefan M. Pfister, Marcel Kool

**Affiliations:** 1 Hopp Children’s Cancer Center (KiTZ), Heidelberg, Germany; 2 Division of Pediatric Neurooncology, German Cancer Research Center (DKFZ), German Cancer Consortium (DKTK), Heidelberg, Germany; 3 Princess Máxima Center for Pediatric Oncology, Utrecht, The Netherlands; 4 Department of Pediatric Oncology/Hematology, Charité University Medicine, Berlin, Germany; 5 Department of Neuropathology, Heidelberg University Hospital, Heidelberg, Germany; 6 Clinical Cooperation Unit Neuropathology, German Cancer Research Center (DKFZ), Heidelberg, Germany; 7 Department of Pediatric Oncology, Hematology and Immunology, University Hospital Heidelberg, Heidelberg, Germany

## Abstract

Embryonal tumor with Multilayered Rosettes (ETMR) is a relatively rare but typically deadly type of brain tumor that occurs mostly in infants. Since the discovery of the characteristic chromosome 19 miRNA cluster (*C19MC*) amplification a decade ago, the methods for diagnosing this entity have improved and many new insights in the molecular landscape of ETMRs have been acquired. All ETMRs, despite their highly heterogeneous histology, are characterized by specific high expression of the RNA-binding protein LIN28A, which is, therefore, often used as a diagnostic marker for these tumors. ETMRs have few recurrent genetic aberrations, mainly affecting the miRNA pathway and including amplification of *C19MC* (embryonal tumor with multilayered rosettes, *C19MC*-altered) and mutually exclusive biallelic *DICER1* mutations of which the first hit is typically inherited through the germline (embryonal tumor with multilayered rosettes, *DICER1-*altered). Identification of downstream pathways affected by the deregulated miRNA machinery has led to several proposed potential therapeutical vulnerabilities including targeting the WNT, SHH, or mTOR pathways, MYCN or chromosomal instability. However, despite those findings, treatment outcomes have only marginally improved, since the initial description of this tumor entity. Many patients do not survive longer than a year after diagnosis and the 5-year overall survival rate is still lower than 30%. Thus, there is an urgent need to translate the new insights in ETMR biology into more effective treatments. Here, we present an overview of clinical and molecular characteristics of ETMRs and the current progress on potential targeted therapies.

## Diagnosis of ETMRs

Embryonal tumor with Multilayered Rosettes (ETMR) is an aggressive, WHO-grade IV, brain tumor that occurs predominantly in infants under the age of 3 years [77, 124]. ETMRs were only recognized as a distinct entity in recent years and since the tumors are clinically highly heterogeneous in terms of location and histology, the disease has not been extensively studied [87]. Previously, ETMRs were classified under the umbrella of primitive neuroectodermal tumors of the central nervous system (CNS-PNETs) [88] and based on morphological patterns different histological variants were recognized, termed Embryonal Tumor with Abundant Neuropil and True Rosettes (ETANTR) [32], Ependymoblastoma (EBL) and Medulloepithelioma (MEPL) [88, 89]. However, both DNA methylation profiling and transcriptome analysis has clearly demonstrated that ETANTRs, EBLs, and MEPLs, all belong to the same molecular entity, collectively named ETMR, and which has been included in the WHO classification of CNS tumors since 2016 [77, 87].

**Fig. 1:**
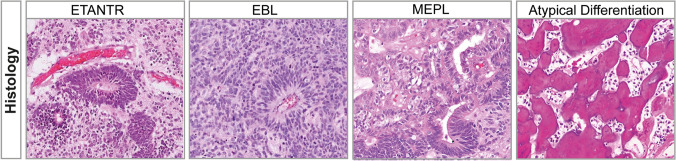
Histology of ETMRs. Each panel shows diverse histological variants of ETMRs with the last panel showing an example of an atypical osteoid differentiation pattern in an ETMR [105]

Even though the histological patterns in ETMRs are diverse, there are characteristic features commonly observed in all ETMRs (Fig. 1). These features include large areas of neuropil, which contains a mixture of unmyelinated axons, dendrites and glial cells, and rosette structures consisting of multilayered mitotically active layers of neuroepithelial cells growing around a lumen [32, 43]. ETMRs with ETANTR histology have large areas of neuropil with dense clusters of small cells that form rosettes, while ETMRs with EBL histology have an overall lower neuropil content but feature large sheets or clusters of poorly differentiated rosettes [32, 77]. MEPL histology is relatively rare but presents as tubular structures resembling the primitive neural tube, and tumors show low levels of neuropil [17]. ETMRs also occasionally present with structures that are atypical for the entity. For instance, there have been reports that ETMRs show a pineoblastoma-like histology and the histology of ETANTR tumors has also been confused with CNS neuroblastoma in the past [43, 80, 124, 126]. Furthermore, there have been reports describing rare instances of divergent differentiation patterns in ETMRs, including osteoid, myeloid, epithelial, mesenchmyal or muscular differentiation [1, 17, 105, 129].

## Molecular diagnosis of ETMRs

Because ETMRs show diverse patterns of histology, the diagnosis currently relies heavily on the identification of molecular characteristics. The first molecular characteristic, described in 2009, was amplification of the 19q13.42 locus that houses one of the largest microRNA clusters in the human genome named *C19MC* [75, 83, 110]. *C19MC* amplification is now considered as the genetic hallmark of ETMRs, present in ~ 90% of all ETMRs regardless their histology (Embryonal tumor with multilayered rosettes, *C19MC*-altered) [80]. The amplification can be identified using fluorescence in situ hybridization (FISH) or copy number profiling with either SNP arrays, DNA methylation arrays or next generation sequencing (NGS) approaches [75, 80]. In 2012, a second molecular characteristic was identified, as it was shown that all ETMRs, regardless of histological variant, stain highly positive for the marker LIN28A, even though this expression is restricted to rosette forming cells [76]. This marker is very useful for the identification of ETMRs, since the widespread LIN28A positivity as seen in ETMRs is very rarely seen in other brain tumor entities, which are either completely negative or only show some focal positivity [76, 114, 124]. Other less specific markers include the expression of nestin and vimentin in rosette forming cells, which lack expression of neuronal and glial cell markers [32, 43]. Such markers, including synapthophysin (SYP), neurofilamentprotein (NFP), and neuronal nuclei (NeuN) [17], are more expressed in the neuropil that may also contain rare populations of cells positive for astrocyte markers such as glial fibrillary acidic protein (GFAP) [87]. Nevertheless, with the development of new molecular methods to diagnose ETMRs it also became clear that not all LIN28A positive tumors that histologically resemble ETMRs have the *C19MC* amplicon [77, 124]. This affects ~ 10% of all cases and even though the *C19MC* amplification, the genetic hallmark of the disease, is absent in those tumors we recently have shown with DNA methylation profiling and transcriptome analyses that these tumors (*C19MC* −) are still molecularly similar to ETMRs having the *C19MC* amplification (*C19MC* +) [80]. Moreover, we have shown that these *C19MC* − ETMRs frequently have biallelic *DICER1* mutations (Embryonal tumors with multilayered rosettes, *DICER1*-altered) [80].

Currently, the WHO classification of histologically diagnosed MEPLs is still ambiguous as MEPLs with *C19MC* amplification are classified as ETMR, but MEPLs that lack the *C19MC* amplification are listed separately as medulloepithelioma [89]. However, when clustering those tumors based on gene expression or DNA methylation profiling they were not found to be molecularly distinct from ETMRs [80, 124]. Furthermore, there are also MEPLs that occur in the eye, named intraocular medulloepithelioma (IO-MEPL), which histologically resemble ETMRs and are positive for LIN28A staining but lack amplification of the *C19MC* locus [62]. However, in contrast to *C19MC* − ETMRs those tumors were found to be molecularly distinct based on DNA methylation profiling. In addition, IO-MEPLs can occur in older patients and overall have a more favorable outcome. Interestingly, IO-MEPLs also frequently harbor *DICER1* mutations, but currently it is unclear to what extend IO-MEPLs differ from ETMRs [74].

## Tumor location

**Fig. 2:**
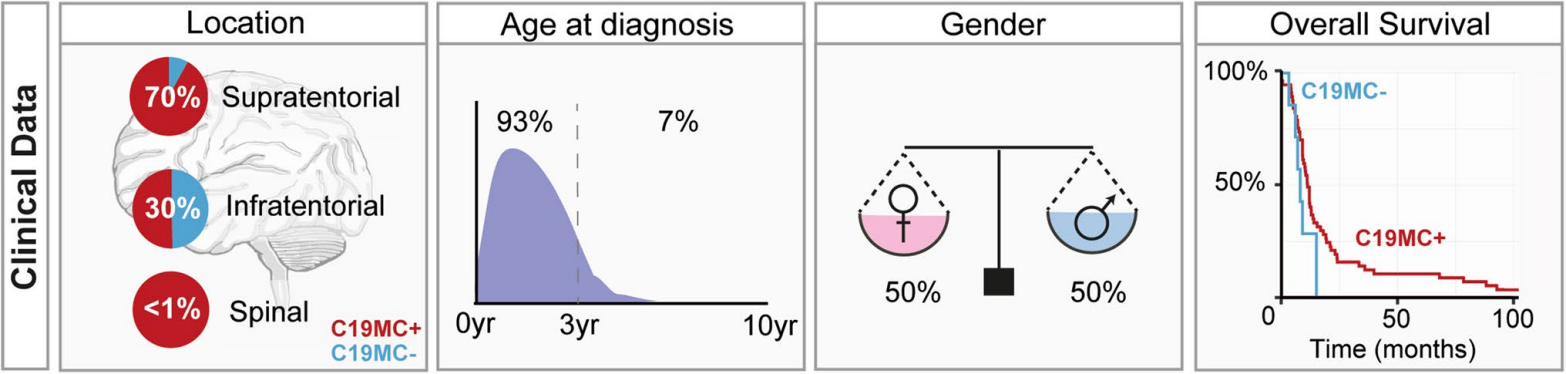
Clinical characteristics of ETMRs. The first panel shows the distribution of ETMRs throughout the brain divided by the presence of *C19MC* amplification, indicating that *C19MC* − ETMRs are more often located infratentorially. The second and third panel show the age and gender distribution of ETMR patients. The few data that is available for *C19MC* − ETMR suggests that there is no difference in age or gender distribution with *C19MC* + ETMRs, which is the reason why they are not visualized separately. The fourth panel shows the overall survival of ETMRs. There is a slight trend that *C19MC* − ETMR patients may do worse, but the difference with the *C19MC* + ETMRs is not significant. *C19MC* + denotes tumors having *C19MC* amplifications, *C19MC* − denotes tumors lacking *C19MC* amplifications

The location of the primary tumor in the central nervous system (CNS) is also heterogeneous. Nearly all tumors reside in the brain, with approximately 70% occurring in supratentorial regions and 30% in infratentorial regions [43, 56, 77] and while there are reports of ETMRs occurring in the spine, those cases are rare [56, 136] (Fig. 2). There is no direct correlation between histological variants and location of the tumor; however, a trend was observed for infratentorial occurrence of *C19MC* − ETMRs, since these tumors often reside near the brainstem [80]. It is not clear whether this may reflect a different cell of origin as the tumors do not seem to separate based on DNA methylation or transcriptome profiling [80].

ETMRs mostly present as large and well demarcated tumors. Using magnetic resonance imaging (MRI), the tumors generally show a heterogeneous signal with frequent diffusion restriction, cystic components, as well as intratumoral hemorrhage [102]. Compared to other CNS-embryonal tumors, the imaging characteristics of ETMRs are similar but the tumor size is overall larger with a mean tumor volume of 115 cm^3^ often spanning multiple lobes [61, 103]. The tumors frequently show very aggressive behavior and rapid growth, even during intensive treatment. Most commonly, tumors progress or recur locally yet distant leptomeningeal metastases may occur, and very rarely extracranial relapses have been observed [77, 119].

## Epidemiology

Reliable epidemiological data that focus on ETMR are not yet available. However, CNS-embryonal tumors, which were previously subsumed within the group of tumors termed CNS-primitive neuroectodermal tumors (CNS-PNET), are diagnosed in roughly 1 per 700,000 children aged 0–4 years [106]. While ETMRs may likely represent a relevant proportion of these tumors in infancy, it has been shown that, historically, frequent misdiagnosis occurred among patients diagnosed with CNS-PNETs [126]. Therefore, molecularly guided specification of diagnosis is needed to reliably determine the incidence of ETMR. The majority of ETMR patients is diagnosed at a very young age, since only 8% of all patients is over 3 years at diagnosis [77]. Initially, in small series there was a minor gender bias reported with a male to female ratio of roughly 1:1.1 [43, 124]; however, in larger cohorts this bias cannot be confirmed and the incidence seems to be equally balanced between males and females [80] (Fig. 2).

## Treatment and management

Presenting symptoms are similar to other malignant brain tumors that occur at a very young age, and are based on the initial location, the size of the tumor, or secondary obstruction of cerebrospinal fluid circulation. Beside symptoms of raised intracranial pressure, paresis, seizures, visual impairments, ataxia, and torticollis may occur [43, 56]. Due to the aggressive nature of the disease, tumors may acutely present as large tumors with short symptomatic interval that may lead to a poor pre- and postoperative status with neurological impairment [92].

Complete resection is often attempted for patients with localized disease, but the prognostic value of a complete resection as first surgical attempt still needs to be clarified. Similar to other malignant CNS tumors occurring at a very young age, gross total resection may be associated to a survival advantage. Nevertheless, surgery of a large tumor at this young age is associated with a high risk for perioperative complications and certain tumor locations preclude complete resection, in particular tumors residing in or near the brainstem [131]. Interestingly, there have been patients reported, for whom a second resection was performed after application of chemotherapy treatment, which led to long-term survival suggesting a possible benefit of secondary resections [3].

Due to the rarity of the disease, no tumor-specific treatment strategy using chemotherapy has been prospectively evaluated for ETMR patients so far. Current treatment strategies are based on prospective trials that enrolled CNS-PNETs and other high-risk CNS-embryonal tumors and evaluated the effectivity of a combined induction chemotherapy and consolidation with high dose chemotherapy followed by autologous stem cell rescue. These strategies contain different combinations of drugs such as Etoposide, Cyclophosphamide, Vincristine, Methotrexate, Cisplatin, Carboplatin, and Thiotepa. Within these trials, improved survival compared to historic controls was reported for patients with CNS-PNET after increased dose intensity treatment, yet there is no data on whether these strategies are effective for ETMRs specifically [25, 33, 38].

Irradiation of ETMR patients is mostly not attempted, since there is no standard treatment protocol for young children that combines craniospinal irradiation with chemotherapy. Preclusion of craniospinal or local irradiation is mainly due to toxicity and associated risk for leptomeningeal spread, as observed in medulloblastoma [6]. Treatment protocols that combine craniospinal irradiation and chemotherapy have been evaluated for ETMR patients, even though these studies only included older patients, which are rare [42, 59]. Despite the associated risks, a survival benefit has been suggested in a retrospective ETMR cohort and a prospective CNS-PNET cohort, likely containing ETMR patients [5, 56]. Also, when evaluating all published cases of surviving ETMR patients, the majority has received irradiation within the treatment course [63]. Nevertheless, the effectivity of frontline irradiation and effectivity of local irradiation for disease control remains to be evaluated in large prospective ETMR specific cohorts.

## Survival and prognosis

Despite intensive and multimodal treatment, the reported outcome is still poor with 5-year overall survival rates between 0 and 30% (Fig. 2). Many patients show aggressive progression of disease, which often is refractory to treatment and only single patients have been described that could effectively be salvaged upon relapse. However, there have also been reports on long-term survivors [17, 31, 32, 42, 43, 56, 77, 79, 92, 124]. While so far outcome cannot be predicted by disease presentation or treatment factors due to the limited size of the cohorts, the reported data suggest a positive prognostic role for absence of metastases, complete resection and the application of dose-intense chemotherapy and/or irradiation. Still, there are patients fulfilling all positive criteria who develop early relapse and succumb to disease, while on the other hand, the few reported survivors include patients with initial metastatic presentation, incomplete resection and non-irradiated patients [42, 56]. Interestingly, neuronal differentiation observed spontaneously or post-treatment seems to be associated with disease stabilization of variable duration and may represent another favorable prognostic factor [3, 31, 79]. Thus far, the histological variant and presence of (specific) copy number aberrations do not seem to be prognostic factors [77], but it is still unclear whether absence or presence of *C19MC* amplifications, size of the tumor, location of the tumor or age of diagnosis may influence outcome.

The reported data strongly suggest that the currently applied regimens do not offer effective disease control for the majority of the patients. On the other hand, the variability in the course of the disease gives room for hope that the aggressiveness of the tumor may be overcome by introduction of molecularly informed, specific drug combinations.

## Genetic aberrations in ETMR

### C19MC

Around 90% of all patients diagnosed with an ETMR have an amplification at 19q13.42 involving the miRNA cluster *C19MC*, which is thought to be the main driver of the tumor [75, 83, 110]. The roughly 100 kb long miRNA cluster *C19M*C possibly encodes 62 functional miRNAs; however, the exact number of functional miRNAs is unknown due to poor conservation of the cluster [11].

**Fig. 3:**
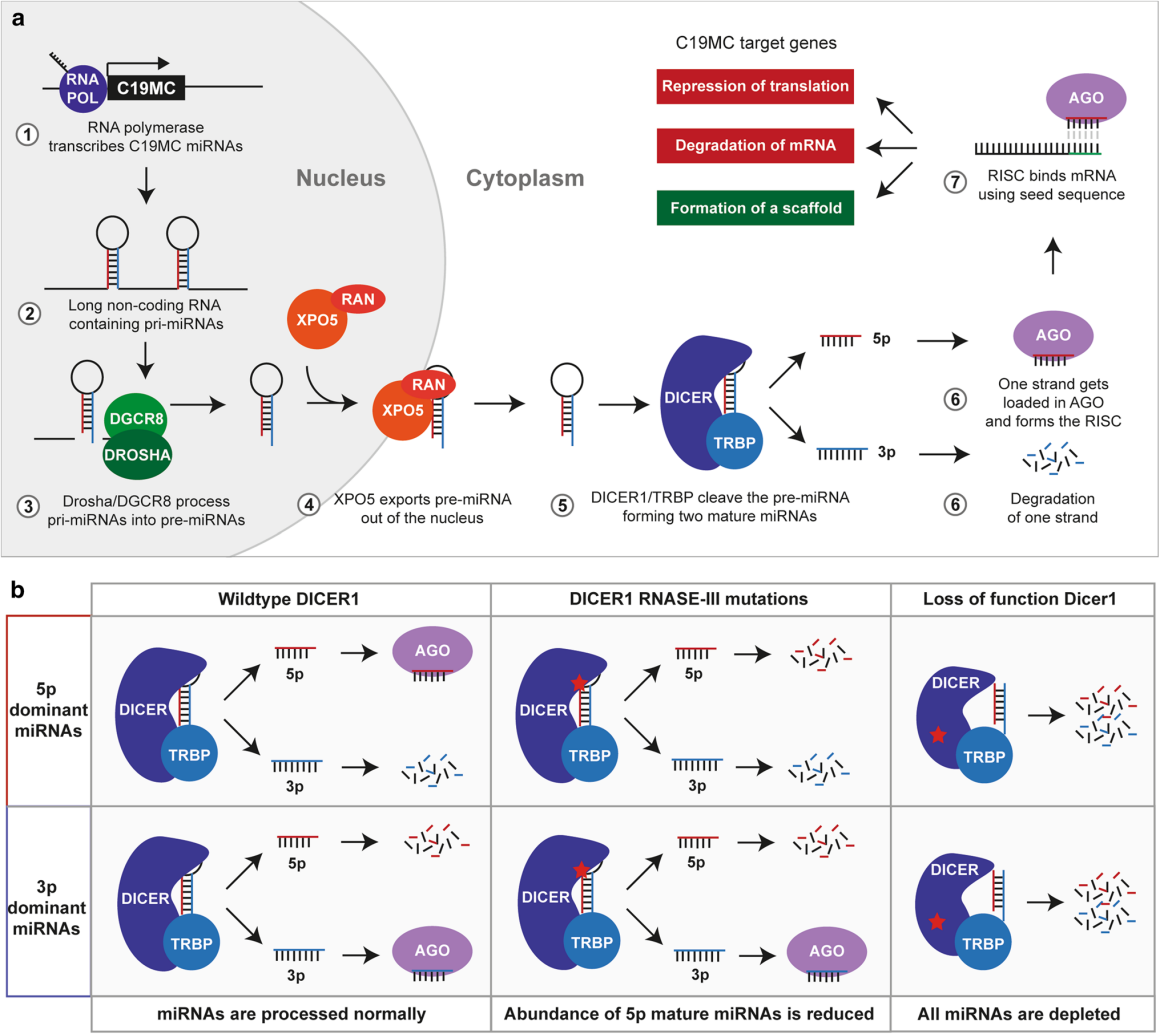
miRNA processing in ETMRs. **a** Overview of miRNA processing and maturation using the *C19MC* miRNA cluster as an example. The effects on *C19MC* target genes are colored based on the result on mRNA translation, green showing an increase and red a decrease in translation. The processing steps shown in this Figure are applicable to all miRNAs and not exclusive to *C19MC*. **b** Overview of different scenarios that mutations in DICER1 have on the abundance of 3p and 5p forms of mature miRNAs

miRNAs are small non-coding RNAs that can be transcribed from separate promotors or processed from long RNA molecules [29, 71]. Functional miRNAs are formed by cleaving RNA molecules, which possibly contain multiple primary miRNAs (pri-miRNA), by a complex of DiGeorge syndrome chromosomal region 8 (DGCR8) and DROSHA [30, 48]. The cleaved products or precursor miRNAs (pre-miRNAs) are then exported out of the nucleus by a complex of exportin-5 (XPO5) and RAS-related nuclear protein (RAN) [30, 104], and subsequently further cleaved by a complex of DICER1 and trans-activation response RNA-binding protein (TRBP) in the cytosol [13, 39]. The resulting 22 bp RNA molecules are the mature miRNAs, which are then loaded onto argonaute (AGO) to form the RNA-induced silencing complex (RISC) [137]. Formation of the RISC allows miRNAs to bind to mRNAs and is generally involved in regulating the abundance of proteins post-transcriptionally, either by inhibiting translation or by destabilization and degradation of an mRNA transcript [58]. However, there are also reports that miRNAs can regulate transcription directly [85, 111] or they may form interactions such as the formation of a scaffold that lead to increased protein abundance rather than a reduction (Fig. 3a) [60, 132].

It is challenging to predict the canonical targets of miRNAs, since the specificity of miRNAs is determined by the “seed” sequence: a small sequence of 6–8 bases [78]. These sequences most commonly bind within the 3′ UTR of a gene, but even when restricted to those regions it still results in hundreds to thousands of predicted sites for each miRNA [22]. This is particularly challenging for *C19MC,* since many individual miRNAs encoded in the cluster have distinct “seed” sequences which further increases the number of potential downstream targets [11, 78].

Validation of possible targets of *C19MC* is difficult, since the cluster only exists in higher primates and is known to have an expression pattern that is restricted to early neural stem cells and the placenta under normal circumstances [11, 81]. The cluster is imprinted and only expressed on the paternal allele when it is active, but in most cells both alleles are epigenetically silenced [9, 101]. In ETMRs, expression of the *C19MC* cluster is likely driven by a translocation and fusion that places the Tweety Family Member 1 (*TTYH1*) gene upstream of the miRNA cluster, followed by amplification of the region [73]. Even though the majority of *C19MC* + cases have a translocation and fusion with *TTYH1*, other fusion partners, including *MYO9B* in a *C19MC* − case and *MIRLET7BHG* in *a C19MC* + case, have been observed*.* It is not clear whether *TTYH1* plays a role in ETMRs, since apart from encoding a chloride anion channel and being expressed in brain, eye, testis and ovary, the function of the TTYH1 protein is currently not well understood [49, 125]. Possibly TTYH1 plays a role in the differentiation of neural stem cells during brain development [70, 143], where *TTYH1* is mainly expressed in neural stem cells, neurons and astrocytes [141]. The cell of origin of ETMRs is currently not known, but there are indications that ETMRs resemble a broad spectrum of developmental stages ranging from neural stem cells and early radial glia to a more differentiated lineage such as astrocytes and neurons based on histology [32, 43, 77], expressed markers [76, 98, 124], and single cell RNA sequencing [64]. It was proposed that loss of *TTYH1* expression during development can lead to loss of *C19MC* expression [64]; however, it remains to be elucidated how this loss in expression can occur.

Despite restricted expression patterns in normal tissue [11, 81], elevated *C19MC* expression has been observed in several other cancers including triple negative breast cancer [65], hepatocellular carcinoma [7, 36], testicular germ cell tumors [35], parathyroid tumors [130], multiple myeloma [12], and mesenchymal hamartoma of the liver [66]. Expression of *C19MC* was in most studies associated to hypomethylation of *C19MC* both at the cluster itself and the upstream region, suggesting that lack of epigenetic silencing can reactivate the cluster [12, 36, 65, 130]. Expression of *C19MC* is also often associated with DNA copy number aberrations of the region, similar to what is seen in ETMRs, as multiple myeloma and triple negative breast cancer show copy number gain [12, 65], while parathyroid carcinoma and hepatocellular carcinoma show amplification of the cluster [7, 130]. Mesenchymal hamartoma of the liver seems to show a different mechanism to activate the cluster: the maternal allele is lost, while the paternal allele, which is actively transcribed, is gained [66]. Nevertheless, a fusion as observed in ETMR has not been reported so far in other tumors.

The function of *C19MC* in other tumors remains elusive, even though *C19MC* expression is generally correlated with a poor outcome and larger tumor size [7, 35, 130]. Nevertheless, it has been shown that several individual *C19MC* miRNAs can increase the efficiency of reprogramming during the generation of induced pluripotent stem cells by inhibiting the epithelial to mesenchymal transition [96, 99]. A similar role was observed in the placenta, where *C19MC* miRNAs prevent trophoblast differentiation through inhibition of WNT signaling [147]. Furthermore, it has been shown that *C19MC* miRNAs can also induce cell proliferation and invasion in trophoblast cells [144]. Similar observations of increased proliferation and invasion were also made in multiple tumor types including breast cancer [57], hepatocellular carcinoma [36], and ETMR [26], suggesting a role for *C19MC* in metastasis and tumor growth. However, the role of *C19MC* miRNAs in tumorigenesis is still ambiguous, since several individual miRNAs have also shown to inhibit proliferation and migration [55, 67]. Furthermore, for *C19MC* miRNAs, having the same “seed sequence” both tumor suppressive and oncogenic functions have been reported [34], suggesting that the downstream mechanisms of *C19MC* are highly context dependent.

### MIR17HG

ETMRs also rarely have amplification of another miRNA cluster on chromosome 13, the *miR-17-92* (MIR17HG) cluster, which was reported in three patients. In two of these patients, the amplification was detected in ETMRs without *C19MC* amplification, while in one case, it co-occurred with *C19MC* amplification [80]. Similar to *C19MC*, the *MIR17HG* cluster has been associated to increased proliferation and invasiveness in multiple cancers and is the first described oncogenic miRNA cluster [94]. Interestingly, the *MIR17HG* miRNA cluster is co-expressed with *C19MC* during placental development [46] and has several “seed” sequences that are identical to mature miRNAs encoded in *C19MC* [78, 91], suggesting that both clusters may be co-regulated and potentially have overlapping functions and targets.

### DICER1 mutations

The second most common genetic event found in ETMRs are biallelic mutations affecting *DICER1*, present in approximately 5% of all ETMR patients and occurring exclusively in tumors lacking *C19MC* or *MIR17HG* amplifications [80, 129]. Mutations affecting *DICER1* show a pattern that is typically found in tumors associated to the *DICER1* predisposition syndrome, having one inactivating germline mutation and one somatic mutation in the RNase III domain [16, 28]. The RNase III domain is involved in cleaving the double stranded precursor miRNAs that forms a hairpin after processing by DGCR8 and DROSHA. This results in either the 3p or 5p arm to be loaded consistently in AGO, while the other is degraded (Fig. 3a) [68]. Based on the structure of DICER1, the catalytic site, where the pre-miRNA is cleaved is mainly formed by the residues S1344 in the RNase IIIa domain, responsible for cleaving the 3p arm, and E1705, D1709 and E1813 in the RNase IIIb domain, responsible for cleaving the 5p arm [133], which are also the residues that are most often mutated in ETMR and other cancers [86, 133]. Mutations at the catalytic domain, irrespective of the affected domain, ultimately leads to an increased loading of 3p forms of mature miRNAs into RISC as the improperly processed 5p form is degraded (Fig. 3b) [2, 133]. In general, this bias in loading 3p miRNAs leads to distinct sites that are targeted as the “seed” sequence differs between the 3p and 5p form [78]. This was shown to mainly affect miRNA clusters that predominantly load 5p forms such as the *let-7* miRNA cluster, while clusters that predominantly load 3p miRNAs are less affected (Fig. 3b) [133].

Cancers associated to the *DICER1* predisposition syndrome, both benign and malignant, primarily have biallelic loss of function mutations and mutations affecting an RNASE III domain of DICER1 (reviewed in [28]). However, there are also tumors such as Wilms tumor or pineoblastoma that recurrently have mutations leading to a complete loss of function of DICER1 or that have mutations in other members of the miRNA processing pathway such as mutations in DROSHA and DGCR8 [82, 84, 109, 128, 135]. These tumors rarely have mutations in the RNase IIIa or RNase IIIb domain of DICER1, suggesting that a general lack of miRNA processing, rather than biased loading of 3p miRNAs, is the driving mechanism in the majority of these tumors [113]. Interestingly, two cases initially histologically diagnosed as pineoblastoma and harbouring RNase IIIb domain mutations in *DICER1* were by DNA methylation profiling reclassified as ETMRs [84]. Together with earlier findings that ETMRs could present with a pineoblastoma-like histology this opens the possibility that the type of aberration affecting *DICER1* could influence what type of tumor develops. Mesenchymal hamartomas of the liver also show *DICER1* mutations affecting the RNase IIIb domain, which are, interestingly, again mutually exclusive with *C19MC* aberrations [4], in line to what is observed in ETMRs [80]. ETMRs were found to be molecularly similar regardless of *C19MC* amplification, suggesting that *DICER1* mutations affecting the RNase III domains and *C19MC* may have common downstream mechanisms. This could potentially explain why other members of the miRNA processing pathway were not found to be mutated in ETMRs [80], since this possibly affects different downstream mechanisms.

### Other recurrent aberrations in ETMRs

Our recent genomic analyses using whole genome and whole exome DNA sequencing have not revealed many other recurrent coding mutations in ETMRs. The most common aberrations include mutations affecting exon 3 of *CTNNB1*, which leads to activation of the WNT signaling pathway by preventing the degradation of the ß-catenin protein [142], identified in 10% of all patients [44, 80, 98], and homozygous or hemizygous *TP53* mutations, occurring in 7% of all patients [80]. Both were identified in *C19MC* + as well as in *C19MC* − ETMRs.

**Fig. 4:**
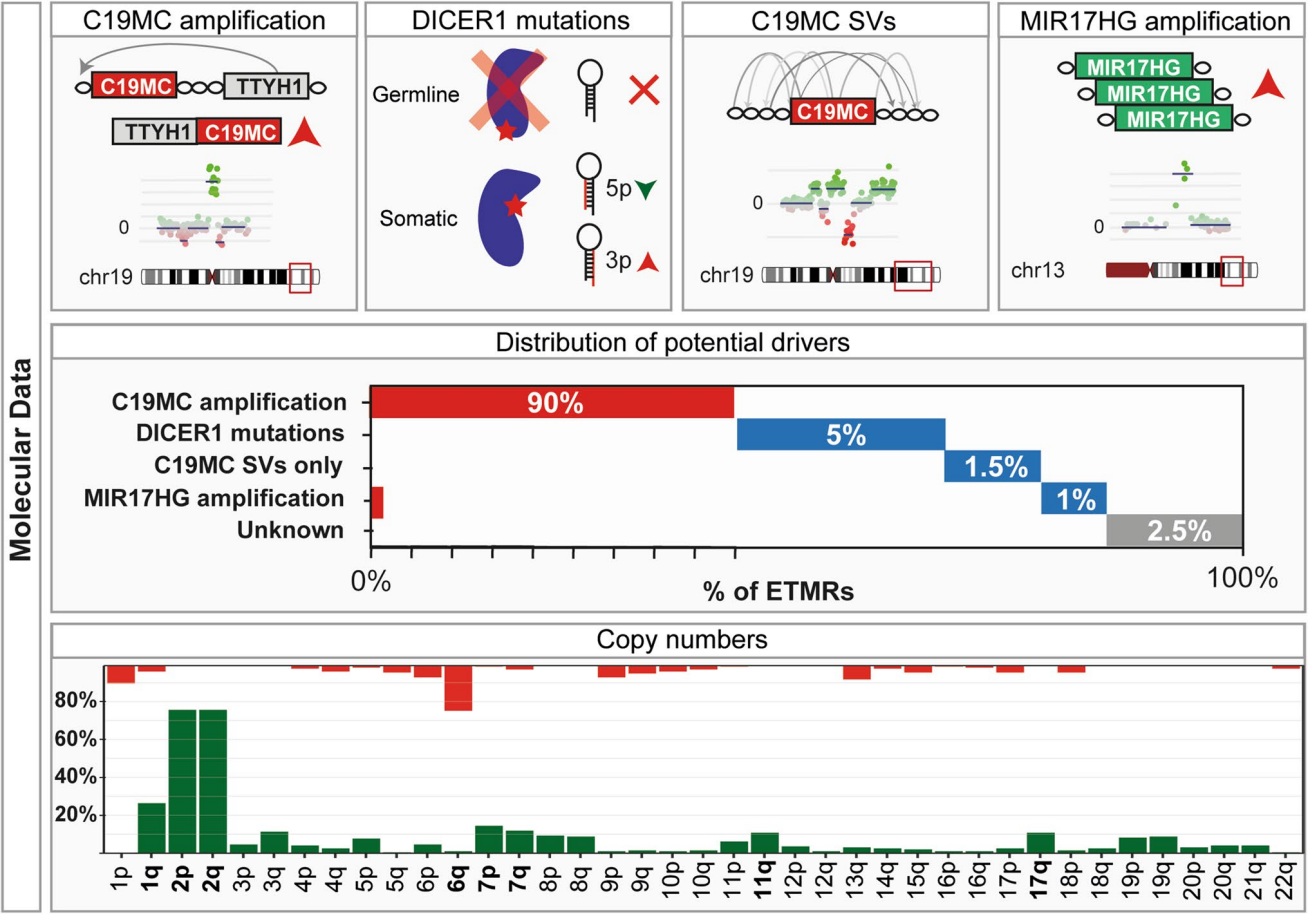
Aberrations found in ETMRs. Figure showing the four identified potential drivers of ETMRs schematically in the top row, the distribution of these aberrations in the second row and the distribution of gains and losses in the last row. In the distribution of potential drivers colors denote C19MC + (red) and C19MC − (blue). In the distribution of copy number aberrations green denotes a gain and red denotes a loss. No significant differences in copy number changes have been observed between C19MC + and C19MC − [80]

ETMRs recurrently have DNA copy-number aberrations such as gain of chromosome 2, reported in approximately 70% of all cases [43, 77, 80, 110]. Other chromosome arms recurrently gained or lost include 1q (gained in ~ 25%), 6q (lost in ~ 20%), 17q (gained in ~ 10%), chromosome 7p and 7q (gained in ~ 10%), 3q (gained in ~ 10%) and 11q (gained in ~ 10%) [77, 80, 120]. Copy number aberrations were often found to be paired with focal chromosomal instability, mainly around *C19MC*, in some cases resulting in more than 100 breakpoints that lead to *TTYH1–C19MC* fusion and amplification [73, 80]. Chromosomal instability in ETMRs is not directly associated to *TP53* mutations as seen in other cancers [115], since only a small subset of cases with high levels of chromosomal instability have *TP53* mutations, suggesting that other mechanisms may play a role in this, such as R-loops (as further discussed below), which have shown to be abundantly present in ETMRs [80]. An overview of genetic aberrations found in ETMRs is given in Fig. 4.

## Active pathways in ETMR

### The LIN28A/let-7 pathway

**Fig. 5:**
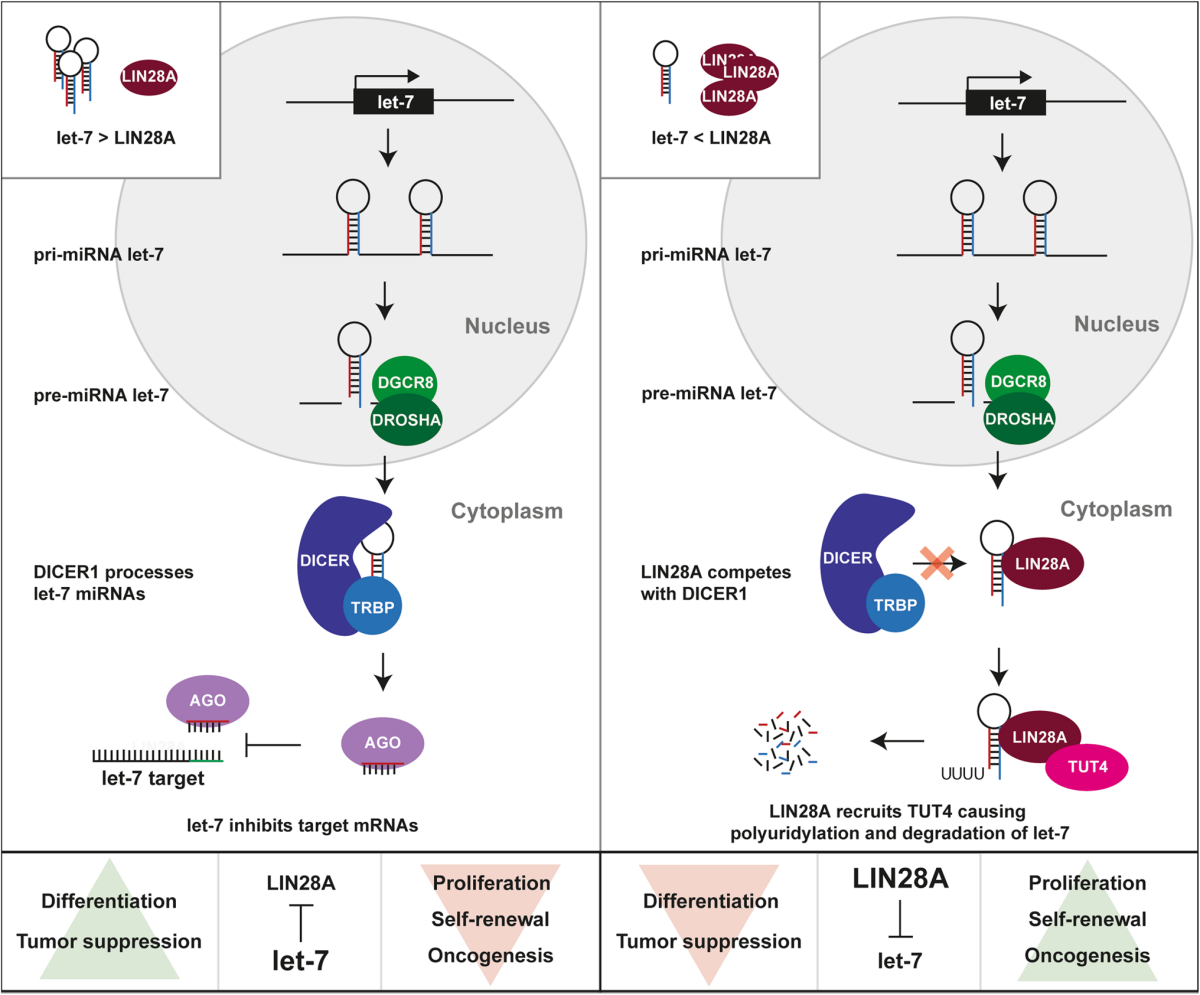
Regulation of let-7 by LIN28A. Schematic overview of miRNA processing of *let-7* and repression of *let-7* targets in the absence (left) or presence (right) of LIN28A. ETMRs have low expression of *let-7* miRNAs and high expression of LIN28A as is shown in the right overview. This pathway is exclusive to *let-7* miRNAs and does not apply to all miRNAs

Apart from mutations affecting the miRNA pathway, all ETMRs have a characteristic high expression of the RNA-binding protein LIN28A [76, 124], known for its role in regulating the *let-7* miRNA family [52]. It has been shown that LIN28A can inhibit maturation of *let-7* miRNAs by binding to the terminal loop of the *let-7* pre-miRNA and recruiting 3′ terminal uridylyl transferase 4 (TUT4) [47, 53]. This leads to polyuridylation, preventing further miRNA processing and eventually leading to degradation of the pre-miRNA, which subsequently leads to reduced levels of mature *let-7* miRNAs in the cell (Fig. 5) [47, 53]. Generally speaking, miRNAs belonging to the *let-7* miRNA family have tumor suppressive functions, while LIN28A and the paralog LIN28B, commonly overexpressed in a diverse spectrum of cancers [8, 149], are considered to be oncogenes. Indeed overexpression of LIN28A or LIN28B is sufficient to induce tumors like neuroblastomas or Wilms tumors in mice [95, 118], but thus far there are no reports showing that they can also induce brain tumors [139]. However, it is clear that LIN28A is important in ETMRs as cell viability is reduced when expression of the gene is silenced [98].

### LIN28A-driven pathways in ETMR

LIN28A is highly expressed in embryonic stem cells and known to be involved in retaining stemness. Retaining an undifferentiated state may be required for ETMRs as well, since ETMRs consist of populations resembling neural stem cells, radial glial cells and more differentiated cells [64, 98]. Since the more undifferentiated cells have higher expression of genes associated to proliferation and have a propensity to grow out upon relapse, they likely play a pivotal role in ETMRs [64, 77, 80]. Currently, it is not clear how undifferentiated cells are retained in ETMRs; however, there are reports suggesting that LIN28A related mechanisms potentially regulate this process. For instance, high mobility group A2 (*HMGA2*), a gene that is highly expressed in embryonic stem cells and involved in the regulation of self-renewal, is a well-documented *let-7* target, and therefore, upregulated when *let-7* miRNAs are repressed through LIN28A [93, 100]. HMGA2 can bind DNA and modify the chromatin state leading to upregulation of multiple pathways involved in oncogenesis including the mTOR pathway, TGF-ß pathway, RAS pathway and cell cycle progression among others [134]. These pathways downstream of HMGA2 and *let-7*, such as the mTOR pathway, are indeed also suggested to play a role in ETMRs as shown by upregulation of the downstream factors IGF2BP1 and IGF2BP2 [15, 76, 123, 150]. HMGA2 also leads to upregulation of NOTCH effectors, including HES5, which play an important role in cell fate decisions of neural stem cells and regulating differentiation timing [107]. Interestingly, both HMGA2 and HES5 are highly upregulated in ETMRs [76] and may possibly underlie the suppression of differentiation in ETMRs.

Other pathways active and suggested to play a central role are the WNT and SHH pathway, which may also contribute to the stemness seen in ETMRs [80, 98]. This is supported by the observation that *CTNNB1* mutations are recurrently observed in ETMRs and a subset of ETMRs show nuclear accumulation of CTNNB1, which is associated with aberrant activation of the WNT pathway [44, 80]. Mutations in SHH pathway genes also occur in ETMRs, but were not found to be recurrent [80, 98]. Furthermore, it has been shown that introducing activating *CTNNB1* and smoothened (*SMO*) mutations in GFAP expressing cells leads to tumor-like structures in the forebrain of mice that histologically resemble ETMRs [98]. In the same study, it was demonstrated that the WNT pathway can be activated downstream of LIN28A through a decrease in *let-7a* miRNAs. However, it is unclear which *let-7a* target modulates the WNT signaling in ETMRs and whether this inhibition is direct or indirect [98]. Possibly, this occurs through upregulation of HMGA2 as well, since several reports have shown a functional link between HMGA2 and WNT signaling [121, 140].

Another major pathway active in ETMRs is the v-myc myelocytomatosis viral related oncogene, neuroblastoma derived (MYCN) pathway. *MYCN* mRNA expression is upregulated in most ETMRs as compared to other pediatric brain tumors, most likely because the gene resides on chromosome 2, which is gained in ~ 70% of all ETMRs [43, 77]. However, focal amplifications of *MYCN* have never been observed in ETMRs [80, 120]. Expression of *MYCN* can also indirectly be regulated by LIN28A as multiple reports have shown that lack of *let-7* miRNAs leads to upregulation of *MYCN* [95, 112, 118]. This can occur either directly, since *let-7* can target *MYCN*, or through activation of ras-related nuclear protein (RAN), which leads to increased transcription and phosphorylation of aurora kinase A (AURKA) and subsequent upregulation of MYCN, as shown in neuroblastoma [118]. Recently, it has been described that ETMRs could indeed be MYCN driven, based on a core regulatory circuit derived from super enhancers, which may explain why ETMRs are overall very aggressive [120].

### Downstream effects of C19MC and DICER1 mutations on the LIN28A/let-7 pathway

The question remains; however, how mutually exclusive genetic aberrations such as *C19MC* amplification or *DICER1* mutations lead to a common downstream mechanism, since the tumors were found to be molecularly similar. A first indication is that both expression of *C19MC* and presence of *DICER1* mutations have been shown to increase the efficiency of reprogramming stem cells [72, 96, 99, 138] and lead to a delay in differentiation in either the placenta, as shown for *C19MC* [147], or globally, in patients affected by the *DICER1* predisposition syndrome [72]. Since reprogramming is also regulated by LIN28A and considering the mutual exclusivity of *C19MC* amplifications and *DICER1* mutations it is attractive to speculate that both recurrent aberrations lead to depletion of *let-7* miRNAs and subsequent upregulation of *let-7* targets.

Currently, a direct link between *C19MC* and LIN28A has not been proven but there is evidence that *C19MC* may affect LIN28A indirectly, for instance by downregulating Tristetraprolin (TTP), a protein that degrades LIN28A [69, 120]. Other factors may also be involved, since many different miRNAs have been shown to target LIN28 directly or indirectly, including *let-7* miRNAs, which generate a negative feedback loop [8, 145], suggesting that a deregulated miRNA processing in general could also underlie changes in the regulation of LIN28A. Furthermore, LIN28 is regulated by pluripotency factors including octamer-binding transcription factor 4 (OCT4), SRY-Box Transcription Factor 2 (SOX2) and NANOG amongst others [18, 24], and considering ETMRs to resemble early neural stem cells the upregulation of LIN28A may also be a secondary event [32, 43, 77].

**Fig. 6:**
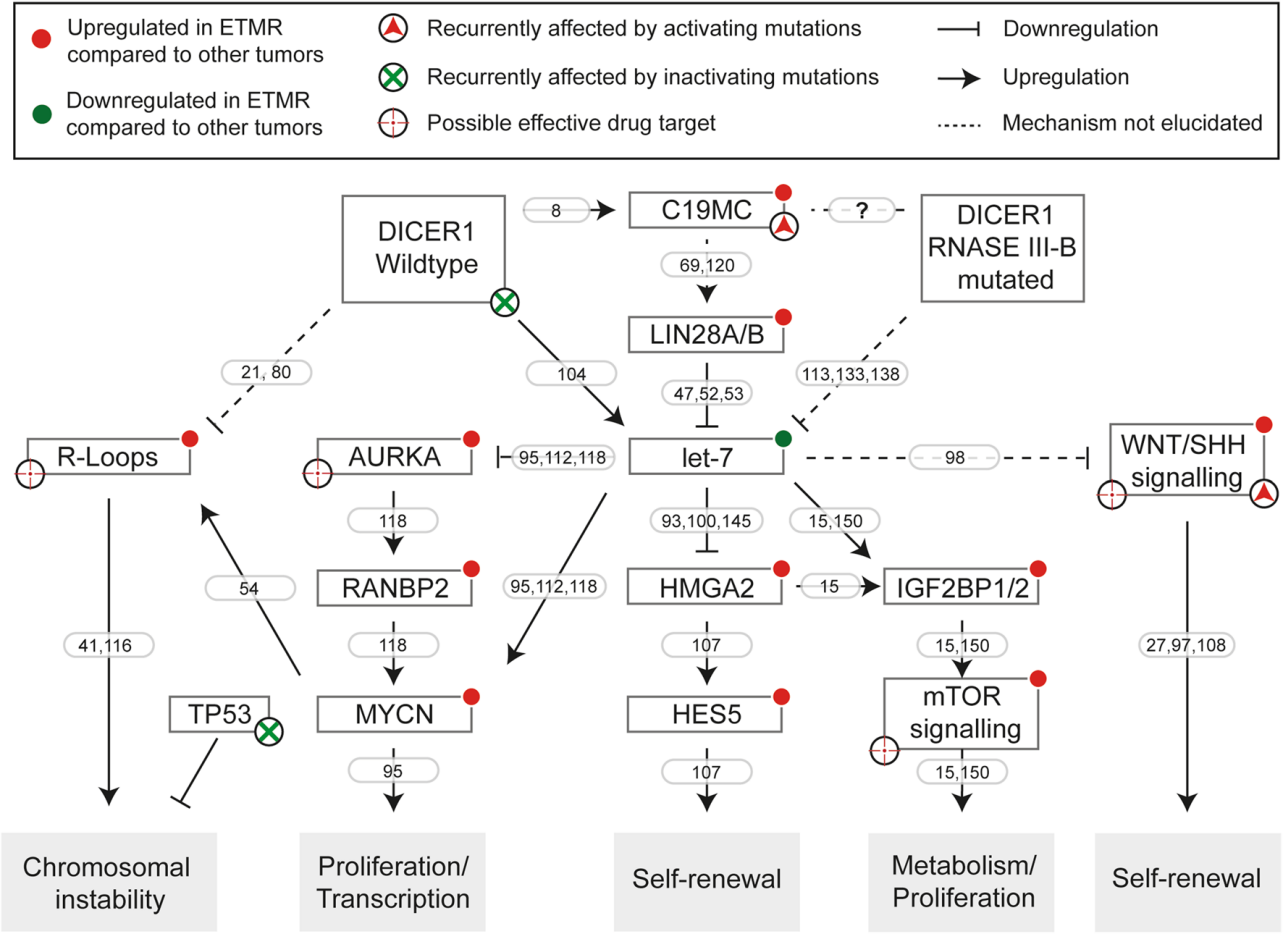
Active pathways in ETMR. Figure illustrating genes and pathways active in ETMR downstream of DICER1 (mutations) and *C19MC* and that have been investigated in ETMRs

DICER1 mutations may also affect LIN28A/let7 signaling. Based on expression profiling in ovarian Sertoli-Leydig-cell tumors, Wilms tumors and Uterine Corpus Endometrial Carcinoma with DICER1 RNase IIIb mutations, *let-7* targets were found among the most significantly upregulated genes compared to other tumors of the same type that lacked DICER1 RNase IIIb mutations [113, 133, 138]. This included factors such as HMGA2 and IGF2BP2 among others, and by re-expressing *let-7* miRNAs the phenotype resulting from DICER1 mutations could be partially rescued [133, 138]. Downstream of DICER1 mutations affecting the RNase IIIb domain, an increase of WNT and MYCN signaling was detected as well, suggesting that DICER1 mutations have similar downstream effects as LIN28A expression [113]. Ultimately, there are many pathways that act downstream of LIN28A and likely drive the tumor (illustrated in Fig. 6), providing new opportunities for rationally designed targeted therapies.

## miRNA processing and chromosomal instability

### The role of miRNA processing factors in DNA repair

Next to its role in regulating oncogenic pathways it is known that the miRNA processing pathway can also be involved in the DNA damage response (DDR). This is illustrated by an impressive number of miRNAs that are differentially regulated after activation of the DNA damage checkpoint [51, 148]. Interestingly, ETMRs recurrently have chromosomal instability and aberrations affecting miRNAs or miRNA processing, while somatic mutations in factors that canonically regulate the DDR, such as TP53, are relatively rare [80].

Furthermore, members of the miRNA processing machinery, in particular DICER1, have also been associated with DDR resulting from replication stress [21, 127]. The importance of DICER1 in preventing replication stress is illustrated by the finding that embryonic lethality occurs when *DICER1* is deleted partially due to increased DNA damage in rapidly proliferative tissues, such as stem cells [14, 127]. Moreover, it has been shown that cells can be sensitized to induced replication stress with a knock down of *DICER1* [127]. It is currently unknown how *DICER1* loss leads to DNA damage upon replication stress; however, it has been postulated that an increase in the level of R-loops, observed after lack of DICER1 function, might play a role [21, 80]. R-loops are structures that form upon stalling of RNA polymerase II and result in a single strand of DNA that hybridizes with a single strand of RNA while displacing the non-template strand of DNA, which remains single stranded. Formation of R-loops can cause or result from a collision of transcription and replication and when R-loops are not properly resolved this can lead to DNA damage and chromosomal instability [41, 116].

One mechanism by which the miRNA processing machinery prevents formation of R-loops is through the regulation of transcription termination. In yeast, it was shown that both dcr1 or ago mutants have an increased read-through of transcription at transcription termination sites that causes replication forks to stall at peri-centromeric repeats [146] and other regions of the genome [21]. This was later shown to cause R-loops to form, likely based on a failure to remove Pol II from the DNA by dcr1 [21]. In mice, it was shown that Dicer1, Ago1 and Ago2 bind at sites of R-loops to facilitate the placement of repressive chromatin marks over termination sites [122], suggesting that Dicer1, Ago1 and Ago2 can prevent chromosomal instability by preventing R-loop formation. Indeed, mouse embryonic stem cells (mESC) having a knockout of *Dicer1* were found to have increased levels of R-loops and DNA damage compared to wildtype mESCs [80]. Currently, it is not clear whether DICER1 mutations affecting the RNase IIIb domain exert a similar function on the formation of R-loops. However, the recruitment of DNA repair factors to sites of DNA damage also relies on phosphorylation of the S1728 and S1852 residues, which affect the RNase IIIb domain and dsRNA binding domain, respectively [19]. This occurs independently of the miRNA biogenesis pathway suggesting that the RNase IIIb domain is also directly involved in maintaining genome stability [37].

The role of *C19MC* in chromosomal instability is still unclear, but since factors other than DICER1 such as DGCR8, DROSHA, AGO1 and AGO2 are also involved in DNA repair, a general deregulation of miRNA processing may underlie the chromosomal instability in tumors lacking DICER1 mutations [40]. Indeed, overexpression of miRNAs can lead to saturation of the miRNA processing machinery and subsequent DNA damage [10, 45]. Furthermore, *C19MC* may also target the DNA repair machinery directly, since expression of *C19MC* in HEK293 cells leads to downregulation of the response to UV radiation and the p53 pathway, which both require the miRNA machinery to repair lesions [20, 23, 96].

## Novel therapeutic leads

Currently, conventional treatment regimens provide only a limited benefit for ETMR patients, warranting the development of novel therapeutic options including rationally designed targeted therapies. Due to the limited number of well-characterized preclinical models for ETMRs it remains challenging to develop and prioritize novel therapeutic options. A screening approach using the BT183 ETMR cell line [123] with a library of 73 different drugs showed that the cell line had increased sensitivity to IGF1R inhibitors, PI3K inhibitors, mTOR inhibitors and topoisomerase inhibitors [123]. Another independent drug screen on the same cell line was performed using 35 different compounds, which confirmed the effectiveness mTOR inhibitors and topoisomerase inhibitors, and resulted in the identification of further compounds such as Actinomycin D, anthracyclines, polo-like kinase inhibitors, aurora kinase inhibitors and the epigenetic drugs decitabine and panobinostat amongst other possibly effective compounds [117]. Activity of several drugs including Topotecan, Volasertib and Actinomycin D was confirmed in vivo using an orthopic xenograft model and combinations with topoisomerase inhibitors and doxorubicin led to a further increase of survival of the mice, yet no long-term cures or disease control [117]. In accordance with the pre-clinical activity of doxorubicin, and actinomycin D, a small number of ETMR patients were also treated according to regimens given to patients with atypical teratoid rhabdoid tumors (ATRT) [50, 117]; however, it remains to be shown that this provides additional benefit.

Recent studies have also proposed other rationally designed treatments based on active pathways rather than a screening approach. One study proposed that SHH inhibitors could be effective in treating ETMRs, based on the capability of forming tumors with ETMR-like histology using activation of the WNT and SHH pathways and a general upregulation of the SHH pathway in ETMRs [98]. Indeed, arsenic trioxide (ATO), which inhibits the SHH pathway, was able to slow the growth of ETMR cells in vitro and in vivo [98]. However, ATO has shown to inhibit other targets as well which lead to differentiation. Therefore, the reduced growth may have been caused by inducing differentiation rather than inhibiting the SHH pathway specifically. Another study proposed to target super enhancers using the bromodomain inhibitor JQ1, since ETMRs were shown to have a MYCN driven super enhancer network and also *C19MC* is likely driven by super enhancers. This has shown to be effective in cell lines but has yet to be tested in vivo [120]. Finally, it was proposed that the chromosomal instability in ETMRs can be used as a therapeutic target based on the presence of high levels of R-loops [80]. Previously, we have shown that R-loops and cell death increased in the BT183 ETMR cell line when treated with topoisomerase inhibitors, which could be further augmented using a combination of PARP inhibitors and topoisomerase inhibitors. Nevertheless, also this potential therapy still needs to be validated in vivo*,* and is additionally depending on whether a good brain penetrance of the drugs can be achieved [80].

## Future perspectives

Since the discovery of the *C19MC* aberration more than a decade ago, there has been an improved understanding of ETMR biology, better diagnostic tools, identification of most driving aberrations and possible downstream targets. In addition, possible vulnerabilities for preclinical and eventually clinical evaluation have been identified. Nevertheless, the survival of ETMR patients has only marginally improved due to the lack of prospective trials and trials evaluating molecularly informed specific drug combinations. Furthermore, due to the limited number of cases and paucity of long-term survivors, the determination of prognostic factors that lead to a favorable outcome is currently not possible. In addition, there is a lack of rigorous pre-clinical testing of drug targets, since there are only few preclinical ETMR models available. This shortage in preclinical models currently impedes the progress towards better therapeutic options and needs to be resolved before moving towards comprehensive clinical evaluation. A better understanding of ETMR biology is required as well; although LIN28 regulated pathways could be promising to target, the connection between DICER1 mutations, *C19MC* amplification and LIN28 expression remains ambiguous.

Nevertheless, the identification of driving events, for instance *DICER1* mutations, and downstream pathways may lead to the development of a more comprehensive set of models that will allow further evaluation of the suggested specific treatments and may possibly identify novel strategies. For instance, it may be possible to target LIN28A directly as was shown to be effective in neuroblastoma [90] or to target epigenetic marks which have been implicated in ETMR pathogenesis [73]. Despite the rarity of the disease, its frequency may increase with improved diagnostics, including molecular classification, as patients were often misdiagnosed in the past [126]. An increased number of identified patients and intense international collaboration will hopefully soon lead to more well-characterized preclinical models and the international set-up of ETMR specific prospective trials. Nevertheless, available data suggest that introduction of preclinically informed specific treatments are on the horizon and with rationally designed targeted therapies and comprehensive pre-clinical testing, it may be possible to eventually improve the outcome of these young patients.
